# The Odontological Society of Great Britain

**Published:** 1887-01

**Authors:** 


ARTICLE IX.

### THE ODONTOLOGICAL SOCIETY OF GREAT BRITAIN.

The first Ordinary Monthly Meeting after the recess was held on the 1st November, Mr. CHARTERS WHITE, M. R. C. S., L. D. S. Eng., President, in the chair.

The PRESIDENT, in a few appropriate sentences, welcomed the members back to the scene of their labors, and hoped that their renewed energies would be brought to bear in furthering the welfare of the Society and the profession generally.

The announcement by the President that it was proposed to honor Mr. George A. Ibbetson by electing him to honorary membership was heartily received, and subsequently the election was unanimously carried by show of hands.

Mr. STORER BENNETT, the Curator, reported several additions to the museum; among them were two specimens sent by Mr. Dunn, of Florence, who thought them two jaws from an Etruscan tomb. In that case they would be highly interesting, as they would probably be about 2,500 years old—not more than that—as prior to that period the Etruscans practiced cremation. The Curator also announced that the Society had purchased the skull of a Manatee.

At the request of the president. Mr. HEPBURN (one of the Secretaries) read a letter from Mr. James Parkinson, the Treasurer, announcing the felicitous terms the presentation to the Society of the portrait of Mr. T. A. Rogers by a few of his friends. The letter dwelt upon the esteem and regard in which Mr. Rogers was held; to the great services he had rendered to the Society and the profession generally during the last thirty years. It referred to his being one of the active Secretaries at the first meeting of the Society in 1859, which office he held until 1861; to the services he had rendered on the Council; to his being unanimously elected President in 1865, and again in 1881. Reference was also made to the various other offices he had filled outside the Society, and to the qualities of kindness and geniality which distinguished him. Mr. Parkinson concluded by trusting that the Society would receive this addition to their walls with a hearty welcome.

The announcement was warmly applauded, and the PRESIDENT, in accepting the presentation officially in the name of the Society, said it was perfectly unnecessary for him to supplement the eulogium contained in Mr. Parkinson's letter, as he felt those remarks really required nothing to be added to them; but, in his capacity as President, he accepted the portrait in their name, and felt they would never look upon that counterfeit presentment without thinking of what represented amiability, dignity and warm-heartedness.

Mr. STORER BENNETT showed models and mentioned a case of imperfect dentition which had been brought to his notice by one of the students of the Dental Hospital (Mr. Colyer.) a short time ago. It occured in a girl, age 16, who had in the lower jaw only two pernament molars, four temporary molars, two canines, and two incisors; the incisors were small wedged-shaped teeth, the jaw itself was small. Although the teeth were deficient in the lower jaw they were very much more so in the upper jaw, which contained only the roots of two canines and the roots of two first permanent molars. Inquiries into her family history failed to reveal anything which would account for the abnormality.

Mr. C. S. TOMES also mentioned a case somewhat on the same lines as the foregoing. In a man, aged about twenty-five, on both sides of the lower jaw and on one side of the upper jaw, the whole molar series were down to the level of the gum. On the right upper jaw the six-year-old molar and the twelve-year-old molar were down nearly to their normal level; but the occassion of his consulting Mr. Tomes was that the twelve-year-old molar was very painful, and on looking at it it seemed as though the wisdom tooth was pressing upon it. On taking hold of it, to his surprise, it came away; it had not, and never had, any roots. He then examined the six-year-old molar on the same side, which was also down to the masticating level, and he thought it was a matter of legitimate inference that none of the molars had any roots. Mr. Tomes thought the point of interest was the fact of three separate checks at three different periods, viz., at the six-year-old molars, the twelve-year-old molars and the wisdom teeth. Mr. Tomes also showed a central incisor which had been placed in his hands by Mr. J. S. Turner; who had extracted it because it was loose. When he took it out he found it had no root; there was no history of an accident, and nothing was left in the socket. so that it had either never formed its root or else it had been absorbed.

Mr. R. H. WOODHOUSE, on examining the tooth, said it had been stopped at the side, and the stopping had been there some years; the stopping on the mesial surface would give considerable pressure and, he suggested, would probably account for development having been arrested.

Mr. COLYER mentioned a similiar case to those referred to by Mr. Storer Bennett and Mr. Tomes, which ocourred in his father's practice. A gentleman, aged about thirty, had, of the permanent set, only the four six-year molars; there were also seven temporary molars, and two malformed incisors, one in each jaw. Beyond suffering from indigestion he was healthly and well developed. The temporary teeth were extracted and artificial dentures fitted. These he had now worn for some years, but no more teeth had made their appearance.

Mr. S. J. HUTCHINSON brought before the notice of the Society one or two little contrivances which he had found useful in his practice. He said that at several previous meetings they had had many interesting hints trom Dr. St. George Elliott—little methods shown which he had found useful in practice. He (Mr. Hutchinson) first alluded to a difficulty which they must all have experienced in mounting gum sections, viz., that of avoiding the dark lines in the joints One way of overcoming this was to put the case in chlorate of lime for six or eight hours, which would entirely clean out the black lines which disfigure gum blocks. Another matter was with reference to pivot teeth. He did not know what the usual practice of members was, but one cuts off a tooth, drills out the fang, and prepares it in such a way that the patient is to come again for a pivot to be fitted in, and goes away with a gap in the mouth. Now, to avoid this gap in the mouth, it was his custom to have a number of what he might call "jerry built" teeth ready, a variety of shades of incisors, laterals, and canines, which might be fitted in temporarily.

Another small hint. Probably they had frequently been troubled in matching artificial teeth where one or two necessary teeth had been wanting; the natural teeth were too dull on the surface or coated with tartar, and nothing would enable them to get a good match. Perhaps it has been the practice to scale the tartar off; well, a little fluoric acid wiped over the artificial teeth would give them just that roughness which would make them undistinguishable from the natural ones The fluoric acid must be kept in a gutta percha bottle.

Lastly, would he mention a way of getting "a dentist's third hand;" and that was a very simple way of fixing to a mouth mirror a small piece of wire which could be pierced into a cork. The cork can then be placed between the patient's teeth and the mirror fixed at any required angle. This second mirror would be found very useful on foggy days in focussing a ray of light.

The PRESIDENT, remarking upon the interesting nature of the hints, said he was glad to have these little "wrinkles" brought forward, believing that they contained the germ of much that was useful. The suggestion with reference to pivots was a very practical one. There was nothing more annoying to a lady patient than to have to proclaim to her friends, by the sudden appearance and disappearance of a gap, that she had taken to artificial teeth. At the same time, he thought that these temporary pivots should be made very secure.

Dr. ST. GEORGE ELLIOTT inquired how Mr. Hutchinson fixed his temporary pivot. A plan which he sometimes adopted as a temporary expedient, in order to ascertain whether a root would bear a pivot or not, was to take an ordinary plate tooth, attach to it with soft solder a German silver pin, insert this into the foramen, and pack amalgam round the head and against the back of the tooth. this could be done in from fifteen to thirty minutes.

Mr. HUTCHINSON replied that he used a hollow pin and floss silk, and took care to make the tooth secure. He had no fear of the patient not returning to have the operation completed; the effect of his temporary stop-gap was not good enough to satisfy a patient even for six weeks.

Mr. F. J. BENNETT said, with regard to jointing continuous gum work, it is the first importance to have them fitting well; but the black line might be prevented by rubbing a little fossiline or osteo over the crack and allowing it to harden. He would like to ask if anyone could tell him how to prevent the black oxide which forms on the cutting surface of American teeth.

Dr. ELLIOTT remarked that it might not be generally known that the characteristic difference between American and English teeth is, that the former are *pressed* into the mould while the latter are *poured* in.

With referencc to another matter, most of the members would be aware that next year there is to be a meeting of the International Medical Congress at Washington, and he had received a letter from Dr. Taft, acting for the Dental Section, stating that they desired to ascertain at the earliest possible moment who would prepare papers and work for the Section of Dental and Oral surgery, and asking him if he would give him the names of 15 or 20 dentists in Great Britain and Ireland who could, and would, prepare papers. It was intended to prepare 10 or 12 operating chairs for the best operators in the world. Benches, lathes, &c., would be provided, and facilities would also be made for various branches of scientific work, operations and treatment of exposed pulps and diseased gums, clinics also would be arranged for.

The PRESIDENT said no doubt many of the younger members would avail themselves of the invitation, although he did not think he should venture to do so himself.

Dr. ELLIOTT: It is very necessary that the Secretary should know in advance the names of the gentlemen who intend to accept the invitation. He would like to say something which was foreign to the subject, viz., that the two colleges, Harvard and Michigan, had lately resolved that the preliminary examination can be held in this country, and, he believed, in any other country.

Mr. WALTER COFFIN said he had been requested by the inventor of a new process—a process for permanently facing vulcanite plates with a metallic surface—to introduce the subject to the notice of the Society. Mr. M. G. Cunningham had specimens which he would show the Society, and from a very long acquaintance with vulcanite work he (Mr. Cunningham) had come to the conclusion that it would be an advantage to place a metallic surface on the rubber rather than by swagging in the usual way.

The method seemed, shortly, to be this: When the ordinary vulcanite piece was so invested in the flask that the flask is open, the unpacked rubber was to be separated from the model and a thin layer of filings or precipitated gold was first spread finely between the model and the vulcanite, then upon either one or the other both a thin layer of calico was placed to prevent the further squeezing of the rubber. The process seemed only to add a few minutes' labor to the ordinary process of making a rubber piece. Mr. Cunningham also wished him to say that a surface properly prepared in this way could be increased by the electro process to any thickness.

The PRESIDENT remarked that he had had the pleasure ofseeing the specimens which Mr. Cunningham had brought, and they certainly added very much to the beauty of the piece, but he was puzzled to understand how an interrupted surface of metal could be made continuous by the bridging over of the intervening spaces of non-conducting material.

Mr. CUNNINGHAM replied that whatever the explanation might be, there was no doubt of the fact that the interrupted surface first obtained could be made continuous by electroplating.

Mr. WALTER COFFIN: The surface of the rubber, when it first comes out of the flask,is of course, not a continuous surface but a mottled surface, therefore practically each deposit would be separated from its neighbor surrounded by rubber; they, therefore, had a discontinuous surface to commence with. The interesting point to him was, that the surface might be deposited upon *electrically*. He had made some experiments himself, with a view of testing this, with the result that he had himself been unable to get any deposit on a surface fairly well covered with *gold filings*, but with a gold *precipitate* he had been able to get a very fair deposit. The only point, to his mind, seemed to be whether or not a sufficient quantity of precipitate could be used, a great deal being lost in the process. He felt bound to say that his experiments were hardly fair to Mr Cunningham, as he had made them quite in an independent way.

Dr. WALKER had tried the process that Mr. Coffin had so clearly described to them, and Mr. Cunningham had kindly called on him and explained the process he was endeavoring to induce them to adopt. He had previously tried electro-plating dentures and, so far as he had gone with it, his first experiments were the same as Mr. Coffin's, and he found in a very short time the deposit stripped from the vulcanite; but when he saw Mr. Cunningham's method, and adopted it, he found he got a clear anchorage for a deposit of gold. He tried an upper piece, and was most careful as to the cleaning of the filings, and, to his great pleasure, it came back beautifully electroed. It had been worn by a patient five weeks and had been returned to him; he had washed it in soap and water, and the deposit gave no evidence of rubbing or wearing off. He had made some three or four cases of this process and, while so far successful, the longest test he had given them was five weeks' wear.

Mr. CUNNINGHAM said the point which appeared to him to be of great importance was to get an electro deposit in the first instance. He was not surprised that an amateur should fail to get a deposit, but he was very much surprised that a professional electro-plater should be unable to do so. The surface merely required to be "quickened" by dipping into a bath of nitrate of mercury, a practice known to all electro-platers.

Mr. COFFIN desired to explain that he particularly instructed his electro-plater to use no preparation to facilitate the deposit, as he understood Mr. Cunningham that his method rendered it unnecessary.

Mr RYMER said he had no doubt many members in former years used gold leaf, as he had done, for coating vulcanite, and it gave a very pretty surface, but the difficulty with the gold leaf was that it got worn off by friction. The question to his mind was, what thickness would be necessary to prevent its wearing off by this method?

Mr. COFFIN had tried years ago every means of coating rubber by the use of plain gold leaf and sponge gold leaf, and he was inclined to think it was not so much *wear* as *pealing away* that had been the difficulty.

In reply to questions by the President, Mr. COFFIN said that it was a very interesting scientific question as to how the conducting is brought about, but he believed the theory was, that the deposit takes place and spreads—like a blush, as it were—from the conducting point over the whole surface. Referring to his experiments, he found that the deposited coat was perfectly inseparable from the surface, and he felt it due to Mr. Cunningham to say that those preparations in which he had succeeded had been done without any preparation of the surface used in the trade such as he spoke of. He had used extremely fine particles, but upon the precipitate the electro deposition was perfect.

Specimens of the work were handed round and admired, and, there being no further discussion,

Mr. WALTER (of Germany) showed and explained a hydraulic press for obtaining well shaped plates, and the meeting separated.—*London Dental Record.*

---

